# Expression of dectin-1 and enhanced activation of NALP3 inflammasome are associated with resistance to paracoccidioidomycosis

**DOI:** 10.3389/fmicb.2015.00913

**Published:** 2015-09-03

**Authors:** Claudia Feriotti, Silvia B. Bazan, Flávio V. Loures, Eliseu F. Araújo, Tânia A. Costa, Vera L. G. Calich

**Affiliations:** Departamento de Imunologia, Instituto de Ciências Biomédicas, Universidade de São PauloSão Paulo, Brazil

**Keywords:** *Paracoccidioides brasiliensis*, dectin-1 receptor, NLRP3 inflammasome, M1/M2 macrophages, innate immunity, resistance to paracoccidioidomycosis, β-glucans

## Abstract

Dectin-1 is a pattern recognition receptor (PRR) that recognizes β-glucans and plays a major role in the immunity against fungal pathogens. *Paracoccidioides brasiliensis*, the causative agent of paracoccidioidomycosis, has a sugar-rich cell wall mainly composed of mannans and glucans. To investigate the role of dectin-1 in the innate immunity of resistant (A/J) and susceptible (B10.A) mice to *P. brasiliensis* infection, we evaluated the role of curdlan (a dectin-1 agonist) and laminarin (a dectin-1 antagonist) in the activation of macrophages from both mouse strains. We verified that curdlan has a negligible role in the activation of B10.A macrophages but enhances the phagocytic and fungicidal abilities of A/J macrophages. Curdlan up-regulated the expression of costimulatory molecules and PRRs in A/J macrophages that express elevated levels of dectin-1, but not in B10.A cells. In addition, curdlan treatment inhibited arginase-1 and enhanced NO-synthase mRNA expression in infected A/J macrophages but had not effect in B10.A cells. In contrast, laminarin reinforced the respective M2/M1 profiles of infected A/J and B10.A macrophages. Following curdlan treatment, A/J macrophages showed significantly higher Syk kinase phosphorylation and expression of intracellular pro-IL-1β than B10.A cells. These findings led us to investigate if the NRLP3 inflammasome was differently activated in A/J and B10.A cells. Indeed, compared with B10.A cells A/J macrophages showed an increased expression of NALP3, ASC, and IL-1β mRNA. They also showed elevated caspase-1 activity and secreted high levels of mature IL-β and IL-18 after curdlan treatment and *P. brasiliensis* infection. Our data demonstrate that soluble and particulate β-glucans exert opposed modulatory activities on macrophages of diverse genetic patterns. Moreover, the synergistic action of dectin-1 and NALP3 inflammasome were for the first time associated with the innate response of resistant hosts to *P. brasiliensis* infection.

## Introduction

Dectin-1 is an innate immune pattern recognition receptor (PRR) that recognizes β-glucans in the cell walls of fungi, and is a crucial receptor for protective immunity against fungal pathogens. Interaction of microbial β-glucans with dectin-1 expressed on myeloid phagocytes [macrophages, dendritic cells (DCs), and neutrophils] activates phagocytosis, reactive oxygen species (ROS) production, synthesis of inflammatory cytokines and chemokines and influences the development of adaptive immunity (Goodridge et al., 2009; Brown, 2011).

Dectin-1 is a type II membrane receptor that contains a single extracellular C-type lectin domain and an unusual immunoreceptor for tyrosine-based activation motif (ITAM) called “hemITAM” in the cytoplasmic tail (Goodridge et al., 2009; Brown, 2011). Besides dectin-1, complement receptor 3 (CR3, CD11b/CD18; Vetvicka et al., 1996), lactosylceramide (Zimmerman et al., 1998) and selected scavenger receptors, including CD36 (Means et al., 2009), have been identified as β-glucan receptors. Curdlan, a particulate β-glucan has been identified as a dectin-1 specific agonist (Goodridge et al., 2009). In contrast, laminarin, a water-soluble linear β-glucan, can bind to dectin-1 without stimulating downstream signaling (Goodridge et al., 2009, 2011), but is able to block the binding of particulate β-glucans such as zymosan to dectin-1 (Goodridge et al., 2011). The immunostimulatory activity of β-glucans has long been recognized, but their diverse structure has also been associated with diverse biological functions (Goodridge et al., 2007, 2009, 2011; Qi et al., 2011). Dectin-1 ligation by β-glucan-containing particles triggers Src/Syk-dependent downstream signals in myeloid cells to activate MAP kinases, as well as NF-κB and NFAT transcription factors (Goodridge et al., 2007, 2011). However, the secretion of IL-1β by β-glucan-activated macrophages was shown to require two steps, a dectin-1/Syk-kinase dependent production of pro-IL-1β and the subsequent activation of NLRP3 (nucleotide-binding oligomerization domain-like receptor P3) inflammasome that employs the adaptor associated speck-like protein (ASC) and caspase-1 to cleave pro-IL-1β into its mature bioactive form (Kankkunen et al., 2010).

*Paracoccidioides brasiliensis*, a thermally dimorphic fungus, is the causative agent of paracoccidioidomycosis (PCM), the most prevalent deep mycosis in Latin America. Our group developed a murine model of PCM where the A/J strain was shown to be resistant and B10/A susceptible to *P. brasiliensis* infection. The protective adaptive immunity of A/J mice is mediated by prevalent type-1 immunity conferred by CD4^+^ and CD8^+^ T cells, whereas susceptibility is associated with anergy of CD4^+^ T cells (Calich et al., 1998, 2008a). Interestingly, the innate immunity developed by these polar mouse strains appears to be paradoxical to the adaptive immunity subsequently developed. The innate immunity of A/J mice is predominantly anti-inflammatory, TGF-β rich, and induces an initial tolerogenic behavior of alveolar macrophages and DCs. However, at the initial phase of infection, high levels of TNF-α were also found in the lungs of A/J mice apparently influencing the development of T cell immunity that expands subsequently (Pina et al., 2008, 2013). Since the early infection, A/J mice develop an elevated number of highly suppressive regulatory T (Treg) cells that tightly control immunity and inflammation, resulting in less destructive disease and improved maintenance of tissue architecture (Chiarella et al., 2007; Felonato et al., 2010). In contrast, the innate immunity of susceptible B10.A mice is pro-inflammatory, IL-12 rich, but the excessive production of nitric oxide and IFN-γ results in total anergy of CD4^+^ T cells and uncontrolled fungal growth, leading to a progressive disease with significant damage to the lungs and dissemination of yeast cells to organs (Calich et al., 1998, 2008a; Chiarella et al., 2007; Pina et al., 2008, 2013; Felonato et al., 2010). Our previous studies with macrophages from resistant and susceptible mice have identified mannosyl-recognizing receptors such as mannose receptor (MR), complement receptor-3 (CR3) and toll like receptor-4 (TLR4) as important pathogen receptors used to sense *P. brasiliensis* infection (Feriotti et al., 2013).

Biochemical studies on the composition of *P. brasiliensis* cell wall have demonstrated that the yeast form is mainly composed of α-glucan, β-glucan, galactomannan, and quitin, while the mycelial form does not contain the outer layer of a-glucan (Kanetsuma and Carbonell, 1970; San-Blas and San-Blas, 1977). Although these early studies have suggested that β-glucans are located in the inner layer of yeast cell walls, and no further studies on the spacial distribution of β-glucans were done, our previous studies suggested that similarly to other fungal infections (Taylor et al., 2007) β-glucans from *P. brasiliensis* yeasts can be recognized by dectin-1. We verified that dectin-1 deficient C57BL/6 mice are highly susceptible to *P. brasiliensis* infection. These mice develop inefficient macrophage activation in the innate phase and impaired T helper-17 (Th17) immune response in the adaptive phase of immunity against *P. brasiliensis* (Loures et al., 2014). We have also recently demonstrated that dectin-1 exerts a synergistic effect with MR and toll like receptor-4 in the differentiation of Th17 and T cytotoxic-17 (Tc17) lymphocytes induced by *P. brasiliensis*-stimulated DCs (Loures et al., 2015). However, studies defining the role of dectin-1 in the experimental model of resistance and susceptibility are still lacking. Therefore, we aimed to characterize the role of particulate (curdlan) and soluble β-glucans (laminarin) in the interaction of *P. brasiliensis* yeasts with macrophages of resistant (A/J) and susceptible (B10.A) mice. We verified that curdlan has a negligible role in the activation of B10.A macrophages but enhances the phagocytic and fungicidal abilities of A/J macrophages by inhibiting their polarization to an M2 profile. On the other hand, laminarin reinforced the respective M2 and M1 profiles of A/J and B10.A macrophages induced by *P. brasiliensis*. Importantly, A/J macrophages that express high levels of dectin-1 showed an enhanced Syk kinase phosphorylation and NALP3 inflammasome activation resulting in increased levels of caspase-1 activity and increased secretion of IL-1β and IL-18. These findings demonstrate that macrophages from resistant mice display a prevalent dectin-1 signaling and NLRP3 inflammasome activation when infected by *P. brasiliensis* yeasts. In addition, our data also show the contrasting stimulatory activities of β-glucans on macrophages of diverse genetic patterns possibly explaining the distinct effects of β-glucans when used in the immunomodulation of infectious or neoplastic diseases.

## Materials and Methods

### Mice

Susceptible (B10.A) and resistant (A/J) mouse strains to *P. brasiliensis* infection were obtained from our Isogenic Unit (Immunology Department of Institute of Biomedical Sciences of University of São Paulo, Brazil) and used at 8–11 weeks of age. Specific pathogen free mice were fed with sterilized laboratory chow and water *ad libitum*. Animal experiments were performed in strict accordance with the Brazilian Federal Law 11,794 establishing procedures for the scientific use of animals, and the State Law establishing the Animal Protection Code of the State of São Paulo. All efforts were made to minimize suffering, and all animal procedures were approved by the Ethics Committee on Animal Experiments of the Institute of Biomedical Sciences of University of São Paulo (Proc.76/04/CEEA).

### Fungus

*Paracoccidioides brasiliensis* (Pb 18 strain), originally isolated from a young patient in 1929, was a gift from Prof. C. Fava Netto. The isolate was maintained by weekly subcultivation in semisolid Fava Netto’s medium at 35°C (Netto et al., 1969). Yeast cells were harvested, washed and adjusted to 8 × 10^4^ cells/mL based on hemocytometer counts. Viability was determined with Janus Green B vital dye (Merck Frankfurter Straße, Darmstadt, GER) and was always higher than 85%. All solutions used to prepare yeast cell suspensions and macrophages were tested for the presence of LPS using the *Limulus amoebocyte* lysate chromogenic assay (Sigma–Aldrich St. Louis, MO, USA) and always showed LPS levels <0.015 EU/mL.

### Phagocytic and Fungicidal Assays

The phagocytic assay was performed as previously described (Feriotti et al., 2013). Thioglycollate-induced peritoneal macrophages were cultivated overnight in Dulbecco’s Modified Eagle’s (Sigma) containing 10% fetal calf serum, 100 U/ml penicillin and 100 μg/ml streptomycin. Macrophages were incubated with or without laminarin (Sigma, MW = 6 kDa) or curdlan (Sigma, MW = 53–2,000 kDa) for 30 min at 37°C in 5% CO2, and then infected with *P. brasiliensis* yeasts in a macrophage:yeast ratio of 25:1 for 2 h. Cells were washed, fixed with methanol, stained with Giemsa (Sigma), and processed by microscopy. For fungicidal assays, after 48 h of co-cultivation supernatants were removed and stored at -70°C. CFU assays were performed to determine the recovery of viable fungi from cell homogenates. Macrophages were untreated or treated with curdlan or laminarin and infected as above described. Two hours after infection, the cultures were gently washed and cultivated for an additional 48 h period. For cytokines and nitric oxide (NO) assays, cell supernatants were removed after 48 h of co-cultivation and stored at -70°C. The cells were then disrupted with distilled water, and 100 μL of cell homogenates were assayed for the presence of viable yeasts.

### Measurement of Cytokines and Nitric Oxide (NO)

Cytokine levels were measured by ELISA (eBiosciences) according to the manufacturer’s protocol. NO production was quantified by a standard Griess reaction (Pick and Mizel, 1981). All determinations were performed in triplicate and are expressed as micromoles of NO.

### Flow Cytometry

For flow cytometry assays A/J and B10.A macrophages were incubated or not with curdlan or laminarin (Sigma) for 30 min at 37°C in 5% CO_2_. Cells were then infected with *P. brasiliensis* yeasts in a macrophage:yeast ratio of 25 1 for 2 h in 24-well culture plates. Cultures were gently washed and cultivated for an additional 4 h period. Supernatants were removed, cultures were washed, and macrophages were detached from plastic plates with fresh cold medium and a rubber cell scraper on ice. Cells were adjusted to 5 × 10^5^ viable cells/ml in staining buffer. Fc receptors were blocked by the addition of unlabeled anti-CD16/32 and then stained with anti-F4/80, CD11b, TLR2, dectin-1, MR, and TLR4 monoclonal antibodies (BD-Biosciences) and fixed with 1% paraformaldehyde (PFA; Sigma). For intracellular staining the macrophages were stimulated for 6 h with 50 ng/mL phorbol 12-myristate 13-acetate, 500 ng/mL ionomycin (Sigma), and monensin (3 mM, eBioscience). After surface staining with anti-F4/80 antibody, macrophages were fixed and permeabilized using the Cytofix/Cytopermkit (BD-Biosciences). Macrophages were stained with anti-Syk and anti-pro-IL-1β antibodies, and samples analyzed by flow cytometry (FACSCanto, BD- Biosciences).

### Quantitative Real-Time PCR

Total RNA was extracted using the TRIzol reagent (Invitrogen) according to the manufacturer’s instructions. The RNA concentrations were determined by spectrophotometer readings. cDNAs were synthesized from 2 μg RNA using the High Capacity RNA-to-cDNA kit (Applied Biosystems) according to the manufacturer’s instructions. Real-time polymerase chain reaction (RT-PCR) was performed using the TaqMan real-time PCR assay (Applied) for suppressor of cytokine signaling-3 (SOCS3), SOCS1, arginase1 (ARG1), NO-synthase2 (NOS2), NALP3, ASC, and IL-1β. Analysis was performed with the MxP3000P QPCR System (Stratagene). All values were normalized to GAPDH, and the relative gene expression was calculated using the Pfaﬄ method (Pfaﬄ, 2001).

### Caspase-1 Activity Assay

Macrophages were detached and adjusted to 5 × 10^5^ viable cells/ml in100 μl of apoptosis wash buffer. Cells were stained with FLICA probe (Immunochemistry Technologies) for 1 h at 37°C in 5% CO2. Active caspase-1 was then measured by flow cytometry.

### Statistical Analysis

Data were expressed as the mean ± SEM. Differences between groups were analyzed by Student’s *t*-test or analysis of variance (ANOVA) followed by the Tukey test using PRISMA 5.04 software (GraphPad). *P*-values under 0.05 were considered significant.

## Results

### Curdlan and Laminarin have Contrasting Effects on the Activation of A/J and B10.A Macrophages upon *P. brasiliensis* Infection

**FIGURE 1:**
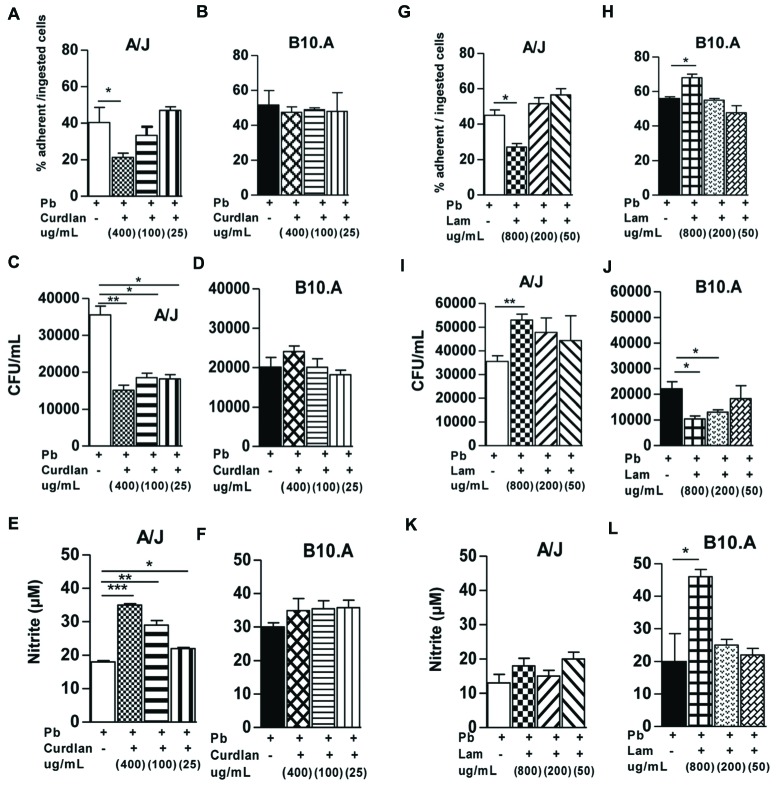
**Curdlan and laminarin treatments have contrasting effects in the activation of A/J and B10.A macrophages. (A,B)** Macrophages cultures performed in round coverslips were treated or untreated with curdlan (400, 100, and 25 μg/mL), or **(G,H)** laminarin (800, 200, and 50 μg/mL) for 30 min and then infected with 4 × 10^4^ viable yeasts (1:25 fungus: macrophages ratio). After 4 h incubation supernatants were aspirated, the monolayer gently washed and the cells stained with Giemsa. An average of 1,000 macrophages was counted and the number of ingested and/or adherent yeasts was determined. **(C,D,I,J)** CFU assays were performed to determine the recovery of viable fungi in cell homogenates. Macrophages were treated by curdlan **(C,D)** or laminarin **(I,J)** and infected as above described. Two hours after infection the cultures were gently washed, cultivated for an additional 48 h period, and the number of recovered viable yeasts measured by a CFU assay. **(E,F,K,L)** Nitric oxide (NO) production was measured in culture supernatants using a Griess reagent. Data are means ± SEM of triplicate samples from two experiments. (^∗^*P* < 0.05, ^∗∗^*P* < 0.01, ^∗∗∗^*P* < 0.001).

First, we determined the effect of curdlan on the phagocytic ability of A/J and B10.A macrophages co-cultivated with *P. brasiliensis* (**Figures 1A,B**). Compared with untreated controls, 400 μg/mL of curdlan reduced the number of ingested or adhered yeasts by A/J macrophages (**Figure 1A**), but curdlan did not impact these processes in B10.A macrophages (**Figure 1B**). When the fungicidal activity was characterized, a diminished recovery of fungal cells was obtained from A/J macrophages with all concentrations of curdlan employed, but such effect was not detected in B10.A macrophages (**Figures 1C,D**). Furthermore, curdlan treatment increased the levels of NO produced by A/J macrophages (**Figure 1E**), but not by B10.A cells (**Figure 1F**).

We have also investigated the effect of laminarin in the interaction between A/J and B10.A macrophages and *P. brasiliensis* (**Figures 1G–L**). Compared with untreated controls, a diminished number of ingested or adhered yeasts by A/J macrophages was observed with 800 μg/mL of laminarin (**Figure 1G**). In contrast, this same laminarin concentration increased the number of ingested or adhered yeasts by B10.A macrophages (**Figure 1H**). In fungicidal assays, laminarin (800 μg/mL) increased the recovery of fungal loads from A/J macrophages, whereas treatment with 800 and 200 μg/mL of laminarin significantly decreased the recovery of viable fungal cells from B10.A macrophages (**Figures 1I,J**). Accordingly, laminarin treatment (800 μg/mL) significantly increased NO production by B10.A, but not A/J macrophages (**Figures 1K,L**). Altogether, our findings demonstrated that curdlan regulates the biological activities of A/J, but not B10.A macrophages. In contrast, laminarin has contrasting effects on A/J and B10.A macrophages: it decreases the phagocytic and fungicidal abilities of A/J macrophages but increases these functions in B10.A cells.

### Curdlan up-Regulates the Expression of Costimulatory Molecules and PRRs in A/J Macrophages, whereas Laminarin Down- and Up-Regulates these Molecules in A/J and B10.A Cells, Respectively

**FIGURE 2:**
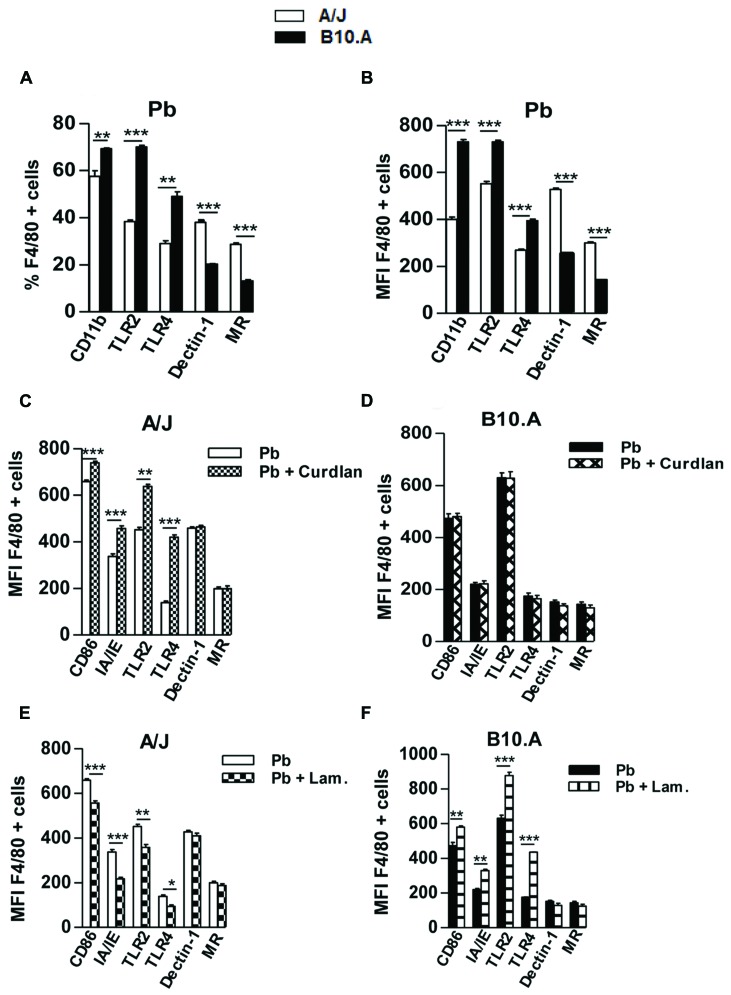
**Curdlan up-regulates the expression of costimulatory molecules and pattern recognition receptors (PRRs) on A/J macrophages, whereas laminarin down- and up-regulates these molecules on A/J and B10.A cell, respectively. (A,B)** Frequency and median fluorescence intensity (MFI) of PRRs expressed by A/J and B10.A macrophages following *Paracoccidioides brasiliensis* infection. The macrophage suspensions were obtained and stained as described in Section “Materials and Methods”. Cells were analyzed by flow cytometry, and the acquisition and analysis gates were restricted to F4/80 labeled macrophages. **(C,D)** MFI of costimulatory molecules and PRRs of curdlan treated (400 μg/mL) or untreated A/J and B10.A macrophages infected by *P. brasiliensis*. **(E,F)** MFI of membrane molecules of laminarin treated (800 μg/mL) or untreated A/J and B10.A macrophages infected by *P. brasiliensis*. Data are means ± SEM of triplicate samples from two independent experiments. (^∗^*P* < 0.05, ^∗∗^*P* < 0.01, ^∗∗∗^*P* < 0.001).

To further characterize the effect of curdlan and laminarin treatments on A/J and B10.A macrophages, the expression of costimulatory molecules and PRRs was assessed in *P. brasiliensis* infected A/J and B10.A macrophages. Compared with A/J macrophages, an increased frequency of positive cells and enhanced expression of CD11b, TLR2, and TLR4 was observed in B10.A macrophages. In contrast, A/J macrophages showed increased levels and frequency of cells expressing dectin-1 and MR (**Figures 2A,B**). An enhanced expression of CD86, IA-IE, TLR2, and TLR4 on A/J macrophages was induced by curdlan treatment; whereas curdlan treated B10.A macrophages did not alter the expression of costimulatory molecules (**Figures 2C,D**). Decreased levels of CD86, IA-IE, TLR2, and TLR4 were detected in laminarin-treated A/J macrophages (**Figure 2E**) while increased expression of CD86, IA-IE, TLR2, and TLR4 was observed in B10.A macrophages (**Figure 2F**).

### Curdlan and Laminarin Treatments Alter the Production of Cytokines

**FIGURE 3:**
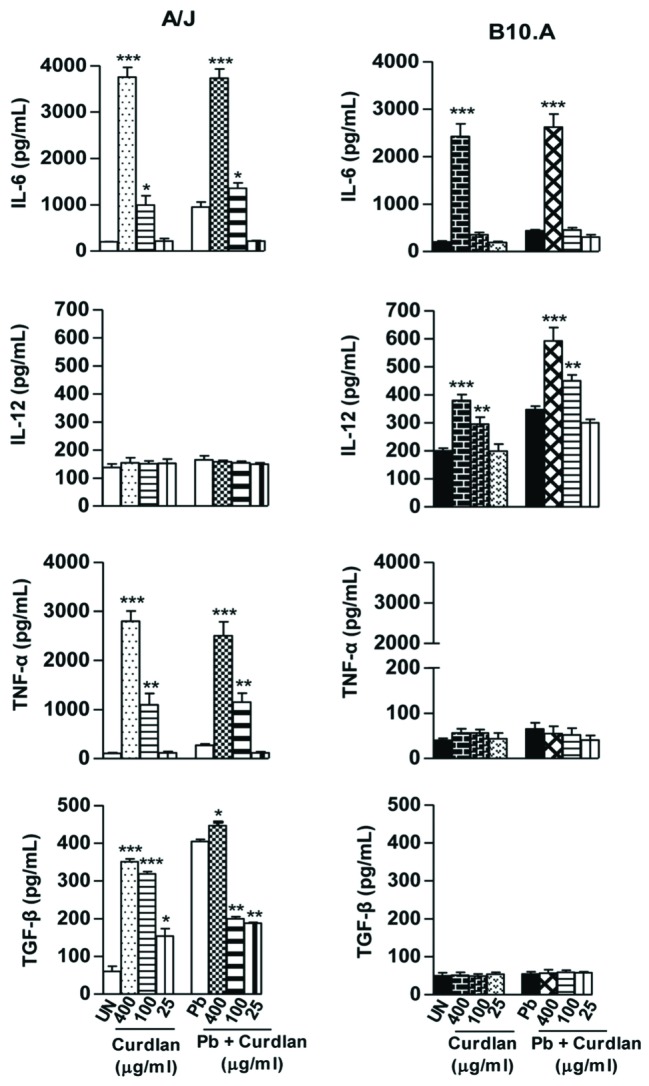
**Curdlan treatment and *P. brasilensis* infection favor the production of TNF-α and IL-6 by A/J macrophages whereas B10.A cells prevalently produce IL-12 and IL-6**. ELISA was used to measure the levels of cytokines in supernatants obtained from fungicidal assays. Macrophages were untreated or treated with curdlan (400, 100, and 25 μg/mL) for 30 min, and cultures infected or uninfected by viable *P. brasiliensis* yeasts (1:25, fungus:macrophages ratio). Co-cultures were maintained during 48 h. Data are means ± SEM of triplicate samples from two experiments. (^∗^*P* < 0.05, ^∗∗^*P* < 0.01, ^∗∗∗^*P* < 0.001).

**FIGURE 4:**
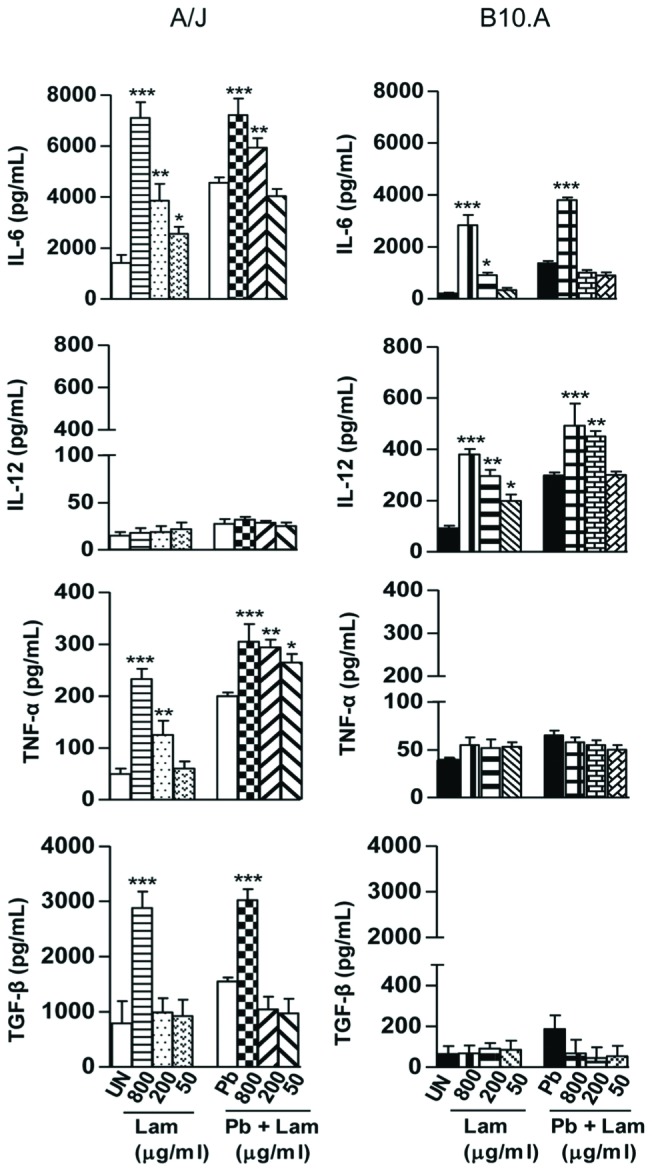
**Laminarin treatment and *P. brasilensis* infection favor the synthesis of TGF-β and IL-6 by A/J macrophages while IL-12 and IL-6 are preferentially synthesized by B10.A macrophages**. ELISA was used to measure the levels of cytokines in supernatants obtained from fungicidal assays. Macrophages were untreated or treated with laminarin (800, 200, and 50 μg/mL) for 30 min, and some cultures infected or uninfected by viable *P. brasiliensis* yeasts (1:25, fungus:macrophages ratio). Co-cultures were maintained during 48 h. Data are means ± SEM of triplicate samples from two experiments. (^∗^*P* < 0.05, ^∗∗^*P* < 0.01, ^∗∗∗^*P* < 0.001).

Supernatants from fungicidal assays were used to determine the effect of curdlan and laminarin treatments on cytokines production by A/J and B10.A macrophages. Curdlan treatment (**Figure 3**) increased the production of IL-6, TNF-α and TGF-β by uninfected or infected A/J macrophages whereas increased IL-6 and IL-12 levels were produced by B10.A macrophages. Of note was the increase of TNF-α (10x) levels induced by the curdlan treatment of A/J macrophages. Laminarin treatment (**Figure 4**) induced a balanced increase in IL-6, TNF-α, and TGF-β by A/J macrophages (1.5–2.0x), and a higher production of IL-6 and IL-12 (3.5–1.5x) by B10.A cells (3.5–1.5x).

### Curdlan Down-Modulates the M2 Profile of A/J Macrophages Whereas Laminarin Reinforces the *P. brasiliensis* Induced M2 and M1 Profiles of A/J and B10.A Macrophages, Respectively

**FIGURE 5:**
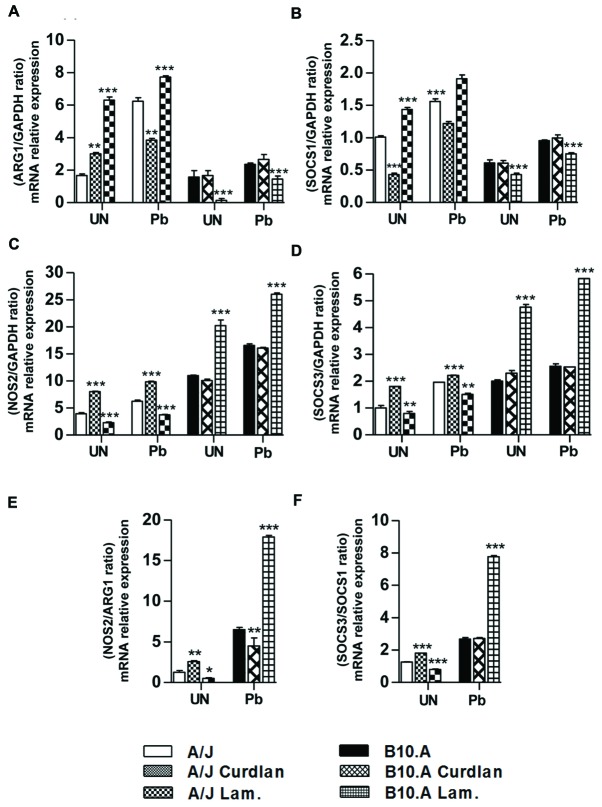
**Curdlan down modulates the M2 profile of A/J macrophages whereas laminarin reinforces the M2 and M1 profiles of A/J and B10.A macrophages, respectively**. Quantitative PCR analysis of **(A)** arginase1 (ARG1), **(B)** suppressor of cytokine signaling-1 (SOCS1), **(C)** NO-synthase 2 (NOS2), and **(D)** SOCS3 mRNA expression. **(E)** NOS2/ARG1 ratios, **(F)** SOCS3/SOCS1 ratios. Macrophages from A/J and B10.A mice were untreated or treated with curdlan (400 μg/mL) or laminarin (800 μg/mL) for 30 min, infected by viable *P. brasiliensis* yeasts (1:25, fungus:macrophages ratio), and cultivated for 12 h. Some cultures were left untreated and uninfected. Total RNA from macrophage cultures was obtained, reverse transcribed, and cDNA amplified. Real-time PCR was performed using TaqMan universal master mix. Amplified products were normalized to the amount of GAPDH products from *in vitro* cultivated macrophages. Data represent the means ± SEM of at least five mice/group of two independent experiments. (^∗^*P* < 0.01, ^∗∗^*P* < 0.05, ^∗∗∗^*P* < 0.001).

The diverse behavior of macrophages induced curdlan and laminarin led us to suppose that these components were modifying the respective M2 and M1 polarization of A/J and B10.A macrophages induced by *P. brasiliensis* infection (Pina et al., 2008; Feriotti et al., 2013; Loures et al., 2014). We therefore determined mRNA expression of M1/M2 gene markers (Gordon and Martinez, 2010) by B10.A and A/J macrophages following laminarin and curdlan treatment and *P. brasiliensis* infection. We verified that curdlan reduced, but laminarin increased the ARG1 and SOCS1 genes expression in infected A/J macrophages. In contrast, curdlan had no effect on ARG1 and SOCS1 mRNA expression of B10.A macrophages but laminarin reduced the mRNA expression of ARG1 and SOCS1 of uninfected and infected B10.A macrophages (**Figures 5A,B**). The **Figures 5C,D** shows that curdlan increased, whereas laminarin reduced the NOS2 and SOCS3 mRNA expression of uninfected and infected A/J macrophages. An opposing effect was observed with B10.A cells. Laminarin increased the NOS2 and SOCS3 expression of uninfected or infected B10.A macrophages but curdlan had no effect on the expression of these M1 markers. The analysis of the M1/M2 (NOS2/ARG1 and SOCS3/SOCS1) ratios (**Figures 5E,F**) expressed by A/J and B10.A cells demonstrates that curdlan increases but laminarin decreases the M1/M2 ratios of A/J macrophages induced by fungal infection. In contrast, laminarin increases the M1/M2 ratios of B10.A macrophages whereas no significant effects were observed in curdlan treated B10.A infected macrophages. Altogether, these findings indicate that curdlan treatment down-regulates the M2 polarization of A/J infected macrophages, but has no important effects in the activation profile of B10.A macrophages. On the other hand, laminarin reinforced the M2 and M1 profiles of A/J and B10.A macrophages, respectively.

### Curdlan Enhances Syk Phosphorylation and Pro-IL-1β Production by A/J but not B10.A Infected Macrophages

**FIGURE 6:**
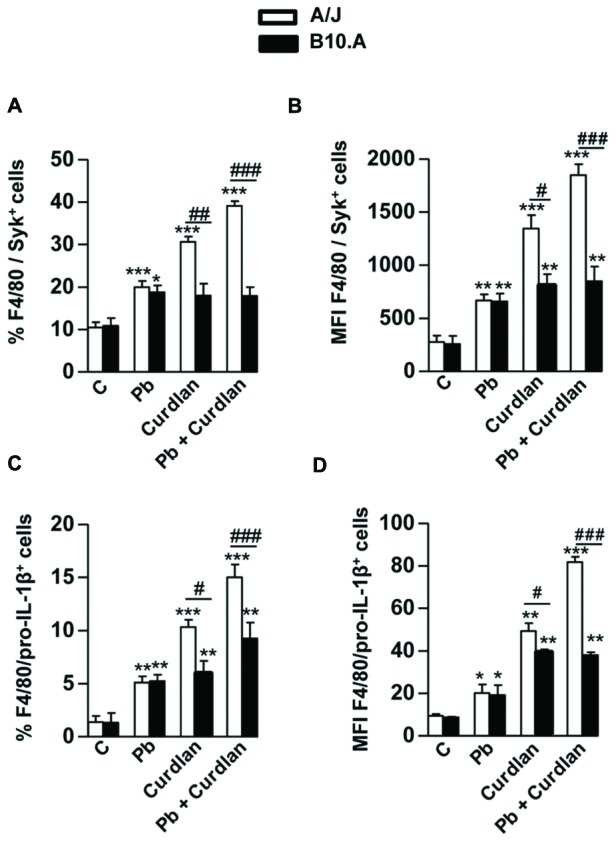
**Curdlan treatment enhances Syk phosphorylation and pro-IL-1β production induced *P. brasiliensis* infection in A/J but not B10.A macrophages. (A)** Frequency (%) of Syk positive macrophages, and **(B)** median fluorescence intensity (MFI) of intracellular Syk phosphorylated protein. **(C)** Frequency (%), and **(D)** MFI of intracellular pro-IL-1β expression by A/J and B10.A macrophages following *P. brasiliensis* infection. A/J and B10.A peritoneal macrophages were treated or untreated with curdlan (400 μg/mL) for 30 min and uninfected or infected with *P. brasiliensis* for 12 h. Cells were analyzed by flow cytometry, and the acquisition and analysis gates were restricted to F4/80 labeled macrophages. A minimum of 50,000 events was acquired on FACScanto II flow cytometer. Data are means ± SEM of triplicate samples from two independent experiments. (^∗^*P* < 0.05, ^∗∗^*P* < 0.01, ^∗∗∗^*P* < 0.001 compared to control; ^#^*P* < 0.05, ^##^*P* < 0.01, ^###^*P* < 0.001, compared to the other mouse strain).

We have further asked whether curdlan and *P. brasiliensis* infection have a different effect on Syk phosphorylation and pro-IL-1β production by A/J and B10.A macrophages. We verified that curdlan treatment or *P. brasiliensis* infection increased Syk (**Figures 6A,B**) and pro-IL-1β (**Figures 6C,D**) expression in both mice strains. However, only A/J macrophages showed an enhanced expression of Syk and pro-IL-1β when cells were stimulated by curdlan or *P. brasiliensis* plus curdlan.

### Curdlan and *P. brasiliensis* Induce Higher NLRP3, ASC, and IL-1β mRNA expression by A/J than B10.A Macrophages

**FIGURE 7:**
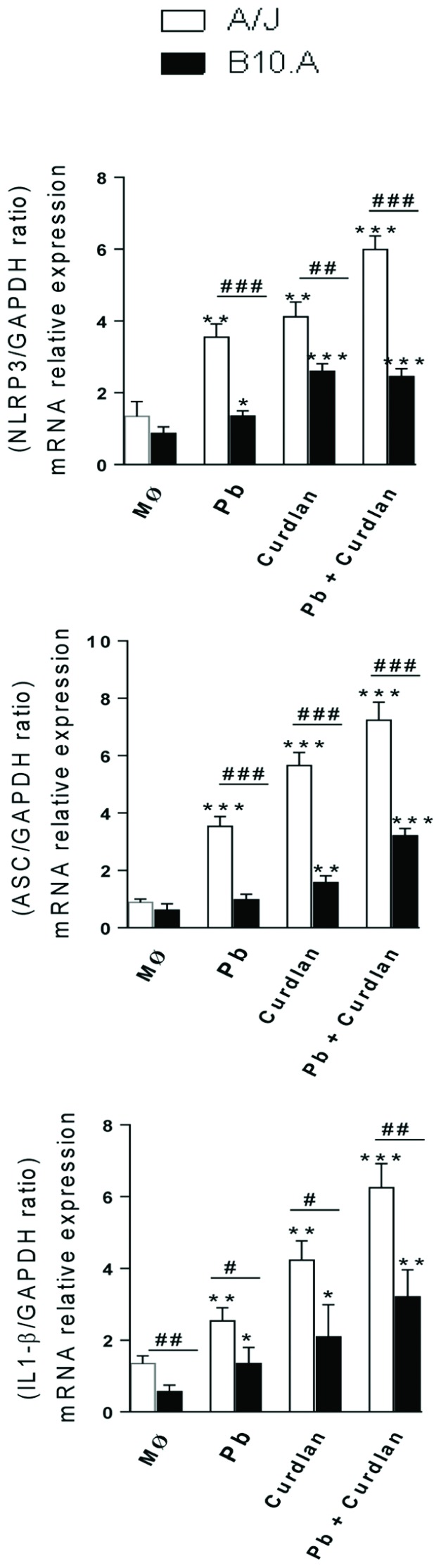
**Quantitative RT-PCR analysis of NALP3 (Nlrp3), ASC (Pycard), and IL-1β mRNA expression**. Macrophages from A/J and B10.A mice were untreated or treated with curdlan (400 μg/mL) for 30 min, infected by viable *P. brasiliensis* yeasts (1:25, fungus:macrophages ratio), and cultivated for 12 h. Some cultures were left untreated and uninfected. Total RNA from macrophage cultures was obtained as described in Section “Materials and Methods”. Data represent the means ± SEM of at least five mice/group of two independent experiments. (^∗^*P* < 0.05, ^∗∗^*P* < 0.01, ^∗∗∗^*P* < 0.001 compared to control; ^#^*P* < 0.05, ^##^*P* < 0.01, ^###^*P* < 0.001, compared to the other mouse strain).

Our current findings demonstrated that dectin-1 is more expressed by A/J macrophages and curdlan exerted a more important effect in the activation of A/J than B10.A cells. This led us to investigate if the NRLP3 inflammasome was differently activated in A/J and B10.A macrophages. Thus, the expression of NALP3, ASC, and IL-1β mRNA was measured by RT-PCR following curdlan treatment and/or *P. brasiliensis* infection of macrophages (**Figure 7**). We verified that all treatments induced in A/J macrophages a significantly higher expression of NALP3, ASC and IL-1β mRNA than in B10.A cells.

### Curdlan and *P. brasiliensis* Induce Higher Caspase-1 Activity and IL-18 Secretion by A/J than B10.A Macrophages

**FIGURE 8:**
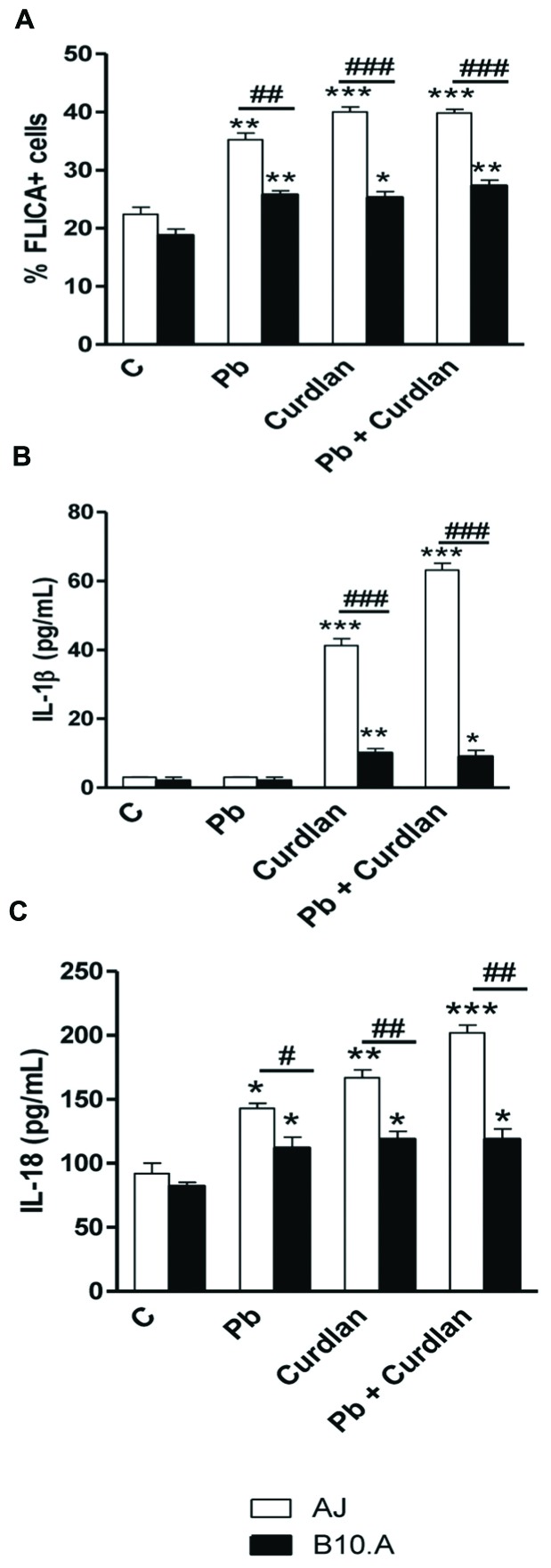
**Curdlan and *P. brasiliensis* induce higher caspase1 activity and mature IL-1β and IL-18 secretion by A/J than B10.A macrophages**. A/J and B10.A macrophages were treated or untreated with curdlan (400 μg/mL) for 30 min, challenged or not with *P. brasiliensis* and cultivated for 12 h. Cells were used to measure caspase1 activity and supernatants to dose mature IL-1β and IL-18. **(A)** Caspase1 activity was evaluated by flow cytometry. Cells were stained with FLICA probe for 1 h and the percentage of FLICA (FAM-YVAD)-positive A/J and B10.A macrophages determined by flow cytometry using a FACScanto II flow cytometer. **(B,C)** Secreted IL-1β and IL-18 were measured by ELISA in the supernatants of curdlan treated and untreated, infected or uninfected A/J and B10.A macrophages. Data are means ± SEM of triplicate samples from two independent experiments. (^∗^*P* < 0.05, ^∗∗^*P* < 0.01, ^∗∗∗^*P* < 0.001 compared to control; ^#^*P* < 0.05, ^##^*P* < 0.01, ^###^*P* < 0.001, compared to the other mouse strain).

To further characterize NLRP3 inflammasome activation, caspase-1 activity and secretion of IL-1β and IL-18 by curdlan and/or *P. brasiliensis* treated and untreated A/J and B10.A macrophages were measured. We verified that all stimuli induced a greater activation of caspase-1 in A/J than in B10.A cells (**Figure 8A**). Interestingly, *P. brasiliensis* infection of A/J and B10.A macrophages resulted in increased secretion of IL-18 but not IL-1β (**Figures 8B,C**). However, curdlan and curdlan plus *P. brasiliensis* led to increased secretion of IL-1β and IL-18 by macrophages of both mouse strains, but these levels were always higher in the supernatants of A/J than B10.A macrophages (**Figures 8B,C**).

## Discussion

Our previous studies have shown that a number of PRRs are involved in the initial sense of *P. brasiliensis* infection. Mannosyl recognizing receptors (MRs, CR3, and TLR4) play a pivotal role in prevalent differentiation of pro-inflammatory, “M1-like,” macrophages by susceptible mice and the healing or “M2-like” macrophages by resistant mice. CR3 is the main PRR engaged by B10.A macrophages that secrete elevated levels of IL-12, however, MR is preferentially used by A/J macrophages that synthesize high levels of TGF-β and TNF-α (Feriotti et al., 2013). Using normal and dectin-1^-/-^ C57BL/6 mice (Loures et al., 2014) we could also demonstrate the protective role of dectin-1 expression by its regulatory activity in the innate and adaptive phases of immunity against *P. brasiliensis* infection.

In this study, we characterized the modulatory effects of curdlan and laminarin, two dectin-1 agonists, on *P. brasiliensis* recognition by macrophages of resistant (A/J) and susceptible (B10.A) mice. These two forms of β-glucans were chosen because the immunomodulatory effects of these components are related to their structure and molecular weight (Brown and Gordon, 2003) remarkably, particulate β-glucans were shown to have a prevalent effect on A/J macrophages that express high levels of dectin-1 on their membranes. In contrast, soluble laminarin was able to activate macrophages from both mouse strains, possibly using multiple PRRs (e.g., CR3, lactosylceramide, CD36) and inducing opposing patterns of macrophages differentiation, M2 for A/J cells and M1 for B10.A macrophages.

The distinct activity of particulate versus soluble β-glucans was clarified by the elegant study of Goodridge et al. (2011). They showed that dectin-1 binds soluble and particulate β-glucans, but only the latter activates phagocytic cells by promoting the formation of phagocytic synapses, exclusion of tyrosine phosphatases, activation of Src and Syk kinases, and subsequent activation of transcription factors (NFkB and NFAT) responsible for phagocytic activity, production of inflammatory cytokines (IL-6 and TNF-α) and activation of massive oxidative burst. On the other hand, laminarin binds, but does not activate Syk and Src kinases neither transcription factors. This distinct pattern of activation was claimed to allow innate immune cells to distinguish between whole microbes from shed microbial components. The complexity of macrophage responses to β-glucans is further increased by its recognition by several PRRs and concomitant activation of TLRs (Calich et al., 2008b; Gordon and Martinez, 2010; Lin et al., 2010; O’Brien et al., 2012; Municio et al., 2013; Loures et al., 2014).

The distinct activities of macrophages here reported were influenced by the different structure of curdlan and laminarin and by the different genetic patterns of mice that constitutively express different levels of PRRs. Indeed, we could demonstrate that curdlan enhances the fungicidal ability, NO production and prevalent secretion of pro-inflammatory cytokines (TNF-α, IL-6, IL-1β, and IL-18) by A/J macrophages, attenuating their “M2-like” profile induced by TGF-β and enhancing the expression of M1 markers, resulting in “M1-like” cells. The pro-inflammatory activity of curdlan was also confirmed by the increased expression of costimulatory molecules, PRRs, and M1 genes markers by A/J macrophages. Of note, was the marked enhancing effect of curdlan treatment on the levels of TNF-α (10x), but not TGF-β (1.1x) induced by *P. brasiliensis*. In contrast, laminarin induced in A/J cells a balanced increase of TNF-α (1.5x) and TGF-β (2.0x).

Laminarin treatment reinforced the respective M2/M1 profiles of A/J and B10.A macrophages induced by fungal recognition. Laminarin decreased the already low fungicidal ability of A/J macrophages that was associated with prevalent TGF-β activity (Pina et al., 2008; Loures et al., 2014). Laminarin has also reduced the levels of membrane PRRs and costimulatory molecules of A/J macrophages, but increased the expression of M2 markers. In contrast, laminarin treated B10.A macrophages showed increased phagocytic and fungicidal abilities, NO, IL-12, and IL-6 production, besides increased expression of PRRs, costimulatory molecules and M1 markers. Using *in vivo* and *in vitro* experiments González et al. (2012) suggested that MyD88, TLR2, TLR4, and dectin-1 do not play a significant role in the recognition of *P. brasiliensis* yeast cells, and these findings are in contrast with our studies that demonstrates the important function of these molecules in PCM (Loures et al., 2009, 2010, 2011, 2014, 2015).

The activity of curdlan was previously shown to exploit two distinct PRRs, the cell membrane dectin-1 and the cytosolic NLRP3 inflammasome. This activation requires the ingestion of β-glucan particles and phosphorylation of Syk kinase, inducing the synthesis of pro-IL-1β/IL-18; the activation of NLRP3 and caspase-1 results in production and secretion of mature IL-1β/IL-18 (Gross et al., 2009; Kankkunen et al., 2010). These pro-inflammatory cytokines belong to the IL-1 family of cytokines and are potent amplifiers of T cell responses. IL-1β was shown to be involved in the differentiation of Th17 responses due its ability of inducing IL-6 and IL-23, whereas IL-18 participates in the production of IFN-γ by NK and T cells contributing to the Th1 response (LeibundGut-Landmann et al., 2007, 2008; Kumar et al., 2009; Agrawal et al., 2010).

The differential expression of dectin-1 by A/J and B10.A macrophages and their distinct response to curdlan stimulation led us to investigate the participation of NLRP3 inflammasome in their responses. It was seen that both, curdlan and *P. brasiliensis* were able to promote the phosphorylation of Syk kinase and induce intracellular pro-IL-1β by A/J and B10.A macrophages. However, an enhancing effect of curdlan in the responses of *P. brasiliensis* infected cells was only observed in A/J macrophages. In addition, under the influence of curdlan or *P. brasiliensis* infection, A/J macrophages expressed higher levels of NLRP3, ASC, and IL-1β mRNA than B10.A cells. The elevated caspase-1 activity and IL-18 secretion by A/J macrophages confirmed their prevalent activation of NALP3 inflammasome. Interestingly, curdlan but not *P. brasiliensis* induced the secretion of IL-1β by A/J and B10.A cells, a finding that was previously described (Tavares et al., 2013) and deserves further investigation. However, an enhancing effect of curdlan on IL-1β secretion was only detected in A/J infected macrophages. Altogether, these results indicate that *P. brasiliensis* and curdlan induce dectin-1 mediated Syk phosphorylation and production of pro-IL-1β. However, NLRP3 inflammasome appears to be more involved in the activation of A/J cells as suggested by the high expression of NLRP3, ASC, and IL-1β mRNA. Moreover, the increased caspase-1 activity and secretion of mature IL-18 reinforced these findings.

Syk kinase, dectin-1, CARD9, and NLRP3 are involved in the host immune response against *Candida albicans, Aspergillus fumigatus, Cryptococcus neoformans* and *P. brasiliensis* (Gross et al., 2006; Yang et al., 2011; Tavares et al., 2013). Syk kinase operates downstream of several immunoreceptor tyrosine-based activation motif (ITAM)-coupled fungal PRR and controls both pro-IL-1β synthesis and inflammasome activation (Hara et al., 2007; Mócsai et al., 2010; Yang et al., 2011). Although pro-IL-1β synthesis is regulated by the Syk-CARD9 pathway, NLRP3 activation occurs through a Syk-dependent but mostly CARD9-independent mechanism (Hara et al., 2007). In addition, NLRP3 activation by fungi requires Syk-triggered ROS generation and K^+^ eﬄux (Underhill et al., 2005). These requirements were also observed in the activation of NALP3 inflammasome of DCs from C57BL/6 mice by *P. brasiliensis* (Tavares et al., 2013).

Our experiments demonstrated that dectin-1 signaling and NLRP3 inflammasome activation play a more important role in the activation of macrophages from resistant than susceptible mice to *P. brasiliensis*. A/J macrophages appear to be more involved in the recognition of particulate β-glucans through dectin-1 whereas the responses of both A/J and B10.A macrophages are mobilized by soluble β-glucans, possibly recognized by other PRRs. We believe that these differences contribute to the opposed pattern of immunity developed by A/J and B10.A mice.

Our previous studies demonstrated that the recognition of *P. brasiliensis* yeasts by A/J macrophages is mediated by several PRRs, some of them inducing TGF-β production that control the initial tolerogenic response of these cells (Pina et al., 2008, 2013; Feriotti et al., 2013). However, the relevant expression and persistent activation of dectin-1 and NLRP3 inflammasome by intact yeast cells and consequent production of IL-6, TNF-α, IL-1β, and IL-18 could surpass the initial tolerogenic effect of TGF-β and ensure the subsequent Th1/Th17 activation that confer immunoprotection to A/J mice (Felonato et al., 2012; Araujo et al., 2014). Furthermore, the M2 inducing activity of soluble β-glucans could contribute to the persistent presence of highly suppressive Treg cells in A/J mice that restrain excessive pro-inflammatory immunity and tissue pathology (Felonato et al., 2012).

The demonstration that soluble and particulate β-glucans exert pro-inflammatory activity on B10.A macrophages, possibly signaling via CR3 or other PRRs that induce elevated levels of IL-6 and IL-12, lead us to suppose that the interaction with both, intact *P. brasiliensis* yeasts and their degradation products, enhances the suppressive pro-inflammatory activity of phagocytic cells involved in the T cell anergy and susceptibility of this mouse strain (Pina et al., 2008, 2013; Nascimento et al., 2012; Feriotti et al., 2013).

## Conclusion

Our findings are important to better understand the immunity against fungal pathogens but also to reevaluate the immunomodulatory functions of β-glucans that were previously shown to be highly dependent on their structure, and here clearly demonstrated to rely on the genetic pattern of individuals. This study opens new perspectives to understand why one single β-glucan preparation can induce in different individuals opposed responses that can result in improvement or worsening of a certain pathological process.

## Conflict of Interest Statement

The authors declare that the research was conducted in the absence of any commercial or financial relationships that could be construed as a potential conflict of interest.
